# Identification of Two Novel Variants in *CRYGD* and *OCRL* Genes in the Chinese Population With Hereditary Congenital Cataracts Using Whole Exome Sequencing

**DOI:** 10.1155/humu/3884362

**Published:** 2026-05-08

**Authors:** Jianlong Zhuang, Nan Huang, Yu′e Chen, Haijuan Lou, Junyu Wang, Wanyu Fu, Chunnuan Chen

**Affiliations:** ^1^ Prenatal Diagnosis Center, Women′s and Children′s Affiliated Hospital of Huaqiao University, Quanzhou Women′s and Children′s Hospital, Quanzhou, China; ^2^ The Teaching and Research Office of Clinical Laboratory Medicine, Quanzhou Medical College, Quanzhou, China, qzygz.com; ^3^ Department of Ultrasound, Quanzhou Women′s and Children′s Hospital, Quanzhou, Fujian, China; ^4^ Research and Development Department, Be Creative Lab Co. Ltd., Beijing, China; ^5^ Department of Neurology, Rare Disease Medical Center, The Second Affiliated Hospital of Fujian Medical University, Quanzhou, Fujian, China, fjmu.edu.cn

**Keywords:** congenital cataract, *CRYGD*, *OCRL*, RNA sequencing, whole exome sequencing

## Abstract

**Background:**

Genetic variants are the leading cause of congenital cataract (CC). To date, numerous genes have been implicated in the development of CC. The objective of the present study was to report two previously unrecognized gene variants associated with CC in two unrelated Chinese families, identified through whole exome sequencing (WES).

**Methods:**

Two unrelated Chinese families affected by CC were recruited. Cytogenetic and molecular genetic analyses were performed using karyotyping, chromosomal microarray analysis (CMA), and WES. In addition, RNA sequencing was conducted to assess differentially expressed genes in affected individuals compared with healthy controls.

**Results:**

Karyotype and CMA elicited none of chromosome abnormalities in both of the families. However, WES revealed a novel missense variant NM_006891.4:c.154 T > C(p.S52P) in the *CRYGD* gene in the proband of Family 1, which was inherited from her mother with CC. In Family 2, a novel frameshift variant NM_000276.4:c.1046dup(p.M349Ifs∗36) in the *OCRL* gene was identified in the fetus via WES, which was inherited from the mother who had CC. RNA sequencing further demonstrated significantly reduced *OCRL* mRNA expression in the fetus compared with age‐matched controls.

**Conclusion:**

The present study reports, for the first time, two novel variants in *CRYGD* and *OCRL* that were identified in the Chinese families with CC. These findings may expand the mutational spectrum of CC and highlight the utility of WES for the genetic diagnosis of patients with CC.

## 1. Introduction

Congenital cataract (CC) is a lens opacity present at birth or during early childhood and represents the leading cause of lifelong visual impairment in children worldwide. CC is both clinically and genetically heterogeneous [1, 2]. Its prevalence is estimated to range from one to six cases per 10,000 live births. A study by Wu et al. [3] reported the highest prevalence in Asian populations (7.43/10,000), followed by the United States and Europe. CC may occur as an isolated ocular anomaly or as part of a multisystem syndrome involving additional organ abnormalities. Although its etiology is multifactorial, approximately 50% of cases are attributable to genetic mutations [4, 5]. In addition, the most common inherited pattern of CC is autosomal dominant (AD) inheritance, but X‐linked and autosomal recessive modes have also been observed [6].

CCs are caused by causative variants in various types of genes, including crystallin genes (e.g., *CRYA*, *CRYB*, and *CRYG*), gap junction protein genes (e.g., *GJA3* and *GJA8*), membrane transport and channel protein genes (e.g., *MIP* and *LIM2*), cytoskeletal genes (e.g., *BSFP1* and *BSFP2*), and transcription factor genes (e.g., *HSF4* and *PITX3*) [7]. Whole exome sequencing (WES) has emerged as a powerful tool in pediatric and prenatal genetic diagnostics. It is increasingly recommended for the etiological investigation of fetuses with structural abnormalities [8, 9]. Previous studies have successfully applied WES to identify pathogenic variants in patients with CC, further supporting its utility in uncovering the genetic basis of the disease [10, 11].

In the present study, WES was applied to two unrelated Chinese families with CC, both showing an AD inheritance pattern. Two previously unreported variants in the *CRYGD* and *OCRL* genes were identified, thereby expanding the mutation spectrum of CC and further illustrating the diagnostic utility of WES in affected families.

## 2. Materials and Methods

### 2.1. Subjects

Two unrelated families affected by CC from Fujian Province, Southeast China, were recruited. The probands and their parents were enrolled for genetic analysis. Prior to sample collection, written informed consent was secured from all participants. Ethical approval was granted by the Institutional Ethics Committee of Quanzhou Women′s and Children′s Hospital (Approval 2023 No. 56). This study was conducted without the use of any AIGC tools.

### 2.2. Karyotype Analysis

Peripheral blood samples (approximately 2 mL from each family member) and amniotic fluid samples (10 mL from the fetus) were collected for karyotype analysis. Following a previously described protocol [12], cultured peripheral blood lymphocytes and amniotic fluid cells were harvested using the Sinochrome Chromprep II automated chromosome harvesting system (Shanghai Lechen Biotechnology Co. Ltd.) and subsequently stained with Giemsa. Karyotypes were described according to the International System for Human Cytogenomic Nomenclature (ISCN 2020) [13].

### 2.3. Chromosomal Microarray Analysis (CMA)

Peripheral blood (3–5 mL) was collected from the proband of Family 1, and amniotic fluid (10 mL) was obtained from the fetus of Family 2. Both samples were subjected to CMA and WES. Genomic DNA was extracted using the QIAamp DNA Blood Kit (QIAGEN, Germany).

CMA was performed using the single‐nucleotide polymorphism (SNP)‐based Affymetrix Cytoscan 750 K array (Life Technologies, United States). Data interpretation of copy number variants (CNVs) was conducted with reference to multiple databases, including the Database of Genomic Variants (DGV), Online Mendelian Inheritance in Man (OMIM), DECIPHER, and PubMed. CNVs were classified according to the standards and guidelines established by the American College of Medical Genetics and Genomics (ACMG) and the Clinical Genome Resource (ClinGen) [14].

### 2.4. WES

Genomic DNA was subjected to quantification, fragmentation, and targeted library preparation, followed by quantification of sequencing libraries. WES was performed on the Illumina HiSeq 2500 platform (Illumina, San Diego, California, United States) with an average sequencing depth exceeding 100×. Data analysis was conducted as previously described [15]. Briefly, variant calling, annotation, and screening were carried out, and allele frequencies (minor allele frequency, MAF < 0.1%) were assessed using public databases, including dbSNP, the 1000 Genomes Project, the Exome Aggregation Consortium, and the Exome Variant Server. Pathogenicity and functional impact of candidate variants were evaluated using the OMIM, ClinVar, Human Gene Mutation Database, and SwissVar databases, following established protocols [15]. Variant classification was performed according to the ACMG guidelines, categorizing variants as pathogenic, likely pathogenic, variants of uncertain significance (VUS), likely benign, or benign [16]. Candidate variants identified through WES were validated by Sanger sequencing.

### 2.5. RNA Sequencing

Total RNA was extracted from aborted fetal tissue of the proband in Family 2 and from three age‐matched controls using TRIzol reagent (Life Technologies, California, United States). RNA concentration and purity were measured using the NanoDrop 2000 spectrophotometer (Thermo Fisher Scientific, Wilmington, Delaware). A total amount of 1‐*μ*g RNA per sample was used as input material for the RNA sample preparations. Sequencing libraries were constructed with the Hieff NGS Ultima Dual‐mode mRNA Library Prep Kit (Yeasen Biotechnology, Shanghai, China), and sample‐specific index codes were incorporated. RNA sequencing was performed on the Illumina NovaSeq platform, generating 150 bp paired‐end reads.

Raw data (raw reads) in fastq format were processed using in‐house Perl scripts. Subsequently, Q20, Q30, GC content, and sequence duplication level were calculated to assess data reliability. The Hisat2 software tool was then employed to map the clean reads to the reference genome. Based on the alignment results generated by Hisat2, the StringTie Reference Annotation Based Transcript (RABT) assembly approach was utilized to construct and identify both known and novel transcripts. For functional annotation of the identified genes, multiple databases were referenced, namely Nr (NCBI nonredundant protein sequences), Pfam (Protein family), KOG/COG (Clusters of Orthologous Groups of proteins), Swiss‐Prot (a manually annotated and reviewed protein sequence database), KO (KEGG Ortholog database), and GO (Gene Ontology). Differential expression analysis between the two groups was conducted with DESeq2. Genes were defined as differentially expressed if they met the criteria of an adjusted *p* value < 0.01 and a fold change ≥ 2, as determined by DESeq2. For the samples without biological replicates, differential expression analysis of two samples was performed using the edgeR. The FDR < 0.01 and fold change ≥ 2 were set as the threshold for significantly differential expression.

## 3. Results

### 3.1. Pedigree Information

**Figure 1:**
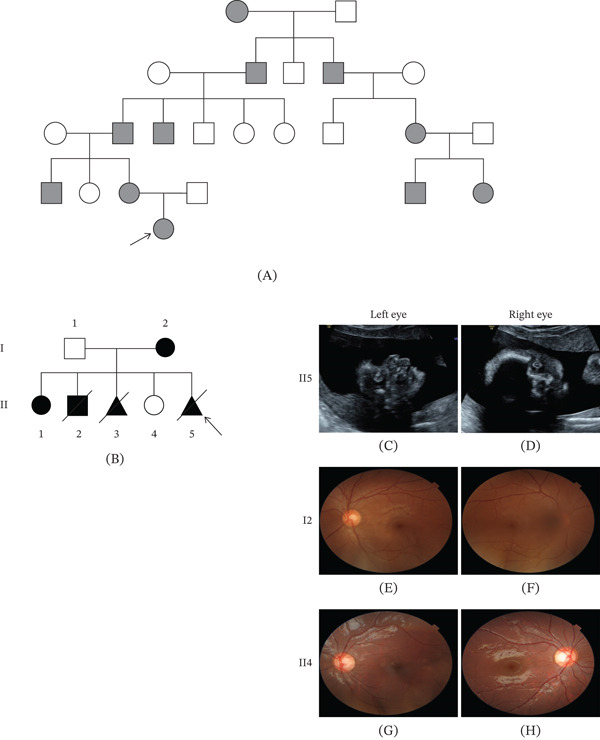
Pedigree information and clinical findings of the enrolled families. (A) Pedigree information of Family 1. (B) Pedigree information of Family 2. (C, D) Enhanced echogenicity of crystalline lens was observed in the fetus of Family 2 (II5). (E, F) Congenital cataract was present in the proband′s mother, with surgery performed on the left side (I2). (G, H) No congenital cataract was detected in the proband′s younger sister (II4).

In Family 1, the proband was a 7‐year‐old female with bilateral CC who was recruited for genetic analysis. The parents reported no consanguinity; however, a family history of CC was present on the maternal side. No abnormalities were detected during the prenatal period. At age 7, the proband exhibited normal developmental milestones, with CC being the only clinical finding. Pedigree analysis, illustrated in Figure 1, suggested a pattern consistent with AD inheritance.

In Family 2, the proband was a 32‐year‐old pregnant woman (G5P2) who presented for clinical consultation and genetic etiology evaluation. The couple reported no consanguinity. The husband was clinically healthy, while the pregnant woman had bilateral CC. As illustrated in Figure 1, the woman had a history of recurrent adverse pregnancies. During the first pregnancy, a female infant with CC was delivered and is currently 14 years old with normal developmental milestones. The second pregnancy resulted in a male infant with multiple severe anomalies, including congenital heart defects, chondrodysplasia, and CC; he died at the age of 1 year. In the third pregnancy, prenatal ultrasound indicated bilateral CC in a male fetus, and the pregnancy was electively terminated. The fourth pregnancy resulted in a healthy female infant with no evidence of cataract or other abnormalities; she is now 4 years old and developing normally. In the most recent pregnancy, increased nuchal translucency (3.1 mm) was observed at 13 weeks of gestation. Doppler ultrasound at 23 weeks revealed polyhydramnios and bilateral CC (Figure 1). The pregnancy was terminated, and amniotic fluid from the aborted fetus was collected for further genetic analysis.

### 3.2. Karyotype and CMA Results

Karyotype analysis and CMA identified no chromosomal abnormalities or pathogenic CNVs in either family.

### 3.3. WES Detection Results

**Figure 2:**
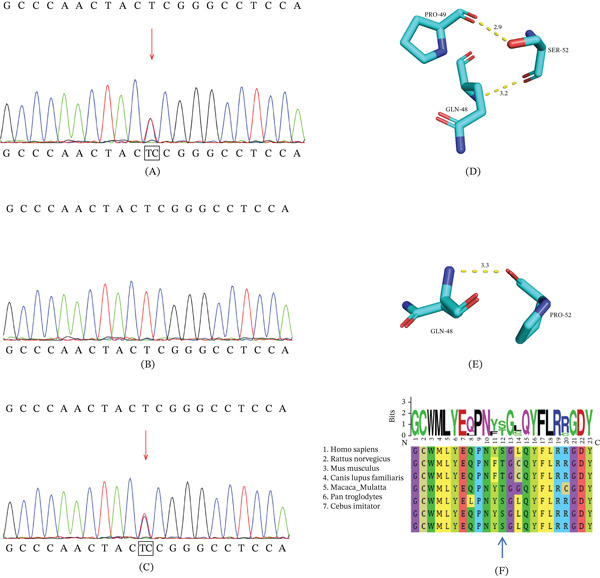
Sanger sequencing and computer‐aided software prediction results for Family 1. (A) Sanger sequencing verified the c.154 T > C(p.S52P) variant of the *CRYGD* gene in the proband, which was inherited from the (C) mother, but was not present in the (B) proband′s father. In the (D) wild‐type, Ser52 forms hydrogen bonds with amino acids at Positions 48 and 49, while (E) after mutation, Ser52 forms hydrogen bonds only with the amino acid at Position 48, suggesting reduced structural stability. (F) Conservation analysis indicated that the c.154 T > C (p.S52P) variant is not highly conserved among species.

Trio‐WES identified a novel missense variant NM_006891.4:c.154 T > C(p.S52P) in the *CRYGD* gene in the proband of Family 1, which was inherited from her mother and cosegregates with CC (Figure 2). The variant c.154 T > C (p.S52P) in *CRYGD* was absent from population databases, including the 1000 Genomes Project, ExAC, gnomAD, and dbSNP, but was reported in ClinVar, where it is classified as likely pathogenic. Conservation analysis indicated that this residue is not well conserved across species (Figure 2). Structural modeling suggested reduced hydrogen bond formation and possible destabilization of the protein structure (Figure 2). According to the ACMG guidelines, the variant was classified as a VUS (PM2_Supporting). No additional variants associated with CC were detected. Further genetic testing of other affected relatives was not possible due to sample unavailability.

**Figure 3:**
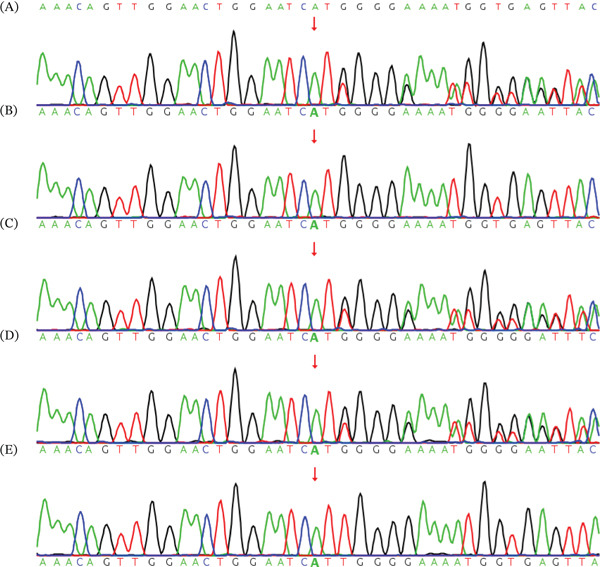
Sanger sequencing results of Family 2. The c.1046dup(p.M349Ifs∗36) variant in the *OCRL* gene identified in the (E) proband, as well as the (C) proband′s mother and (A and D) both sisters, was verified by Sanger sequencing. However, the variant was absent in the (B) proband′s father.

In Family 2, trio‐based WES identified a novel hemizygous variant NM_000276.4:c.1046dup(p.M349Ifs∗36) in the *OCRL* gene in the fetus, consistent with an X‐linked recessive inheritance pattern. The variant was also detected in the proband′s mother and both elder sisters, as confirmed by Sanger sequencing (Figure 3). This frameshift duplication was absent from population and disease databases, including gnomAD and ClinVar. Bioinformatic predictions indicated that the variant would trigger nonsense‐mediated decay, leading to loss of gene function. Based on ACMG criteria, the variant was classified as pathogenic (PVS1 + PP4 + PM2_Supporting + PS3_Supporting). No other variants relevant to CC were identified. The two previously affected male family members were unavailable for molecular genetic testing.

### 3.4. Novel *OCRL* Variant Result in Downregulation of OCRL Expression

To explore the potential pathogenic mechanism of the novel *OCRL* variant c.1046dup(p.M349Ifs∗36), RNA sequencing was performed using fetal tissue from Family 2 and age‐matched controls. A total of 2875 differentially expressed genes were identified. Notably, a significant downregulation of *OCRL* expression was observed in the fetus (File S1), consistent with a loss‐of‐function mechanism underlying the development of Lowe syndrome. Interestingly, a remarkable decreased expression level of *PITX3* was also observed in the fetus (File S1).

**Figure 4:**
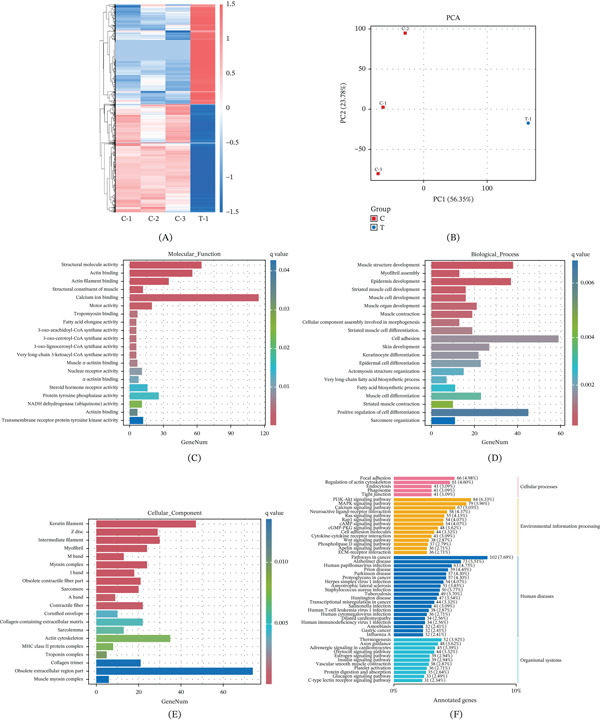
RNA sequencing analysis results for the fetus of Family 2. (A) Heatmap showing significantly upregulated and downregulated genes in the fetus carrying the *OCRL* c.1046dup (p.M349Ifs∗36) variant compared with age‐matched controls. (B) Principal component analysis (PCA) using the positive sample and the controls. (C, D, E) Gene Ontology (GO) enrichment analysis of differentially expressed genes: (C) molecular function, (D) biological process, and (E) cellular component. (F) KEGG pathway analysis showing enrichment in cellular and environmental information processing pathways, including focal adhesion, regulation of the actin cytoskeleton, endocytosis, tight junctions, PI3K–Akt signaling, and cell adhesion molecules.

Functional enrichment analysis was performed on the differentially expressed genes. GO analysis revealed enrichment in molecular functions such as structural molecule activity, actin filament binding, and actin binding. Enriched biological processes included muscle structure development, cell adhesion, and myofibril assembly. In terms of cellular components, genes were enriched in keratin filaments, intermediate filaments, and myofibrils (Figure 4). KEGG pathway analysis demonstrated enrichment in cellular and environmental information processing pathways, including focal adhesion, regulation of the actin cytoskeleton, endocytosis, tight junctions, PI3K–Akt signaling, and cell adhesion molecules (Figure 4).

## 4. Discussion

In the present study, WES technology was applied to investigate gene variants in two unrelated Chinese families with CC. In Family 1, WES identified a novel missense variant, NM_006891.4:c.154 T > C (p.S52P), in the *CRYGD* gene, which may be associated with nonsyndromic cataract. In Family 2, a novel hemizygous frameshift variant, NM_000276.4:c.1046dup (p.M349Ifs∗36), was identified in the *OCRL* gene, which is predicted to cause Lowe syndrome.

Crystallins, including *α*‐, *β*‐, and *γ*‐crystallins, represent the principal structural proteins of the lens, accounting for more than 90% of total lens protein content. Among them, *γ*‐crystallins contribute approximately one‐third of the total, with *γ*D‐crystallin (encoded by *CRYGD*) comprising nearly 25% of crystallins in the human lens [17, 18]. Proper spatiotemporal organization of crystallins is critical for lens transparency, and pathogenic variants in crystallin genes account for a significant proportion of CC cases. Previous studies have demonstrated that mutations in *CRYGD* are frequently implicated in CC. For instance, molecular genetic screening of 218 CC patients revealed *PAX6*, *GJA8*, and *CRYGD* as the three most commonly affected genes [19]. Notably, the majority of reported *CRYGD* mutations are missense variants, which can disrupt hydrogen bond formation, reduce solubility, or destabilize the three‐dimensional structure of *γ*D‐crystallin [20]. The P24T mutation, a recognized hotspot in *CRYGD*, has been shown to markedly reduce protein solubility and is strongly associated with congenital coralliform cataracts [21–23]. In the present study, a novel missense variant c.154 T > C(p.S52P) in the *CRYGD* gene was identified in the patients of Family 1. Structural modeling indicated reduced hydrogen bond formation and potential destabilization of the protein structure. Although this variant is recorded in ClinVar and classified as likely pathogenic, it has not been previously described in the literature. Notably, a different substitution at the same residue, c.155C > G (p.Ser52Trp), was previously reported in a CC patient, highlighting the functional importance of this site. Collectively, these findings suggest that the c.154 T > C (p.S52P) variant in *CRYGD* may be responsible for the presence of CC in Family 1.

Syndromic cataracts are frequently associated with systemic abnormalities, such as those observed in Down syndrome, Wilson′s disease, and other multisystem disorders [24]. Loss‐of‐function variants in the *OCRL* gene cause Lowe syndrome, an X‐linked recessive disorder characterized by CC, central hypotonia, intellectual disability, and renal Fanconi syndrome [25]. Deficiency of *OCRL* can also result in Dent Disease 2, a milder condition that presents primarily with renal Fanconi syndrome in the absence of extrarenal manifestations. Although Lowe syndrome is classically X‐linked recessive, most female carriers develop CC, whereas some remain unaffected, reflecting phenotypic heterogeneity [26]. In Family 2 of this study, trio‐WES identified a novel hemizygous variant in *OCRL* (c.1046dup, p.M349Ifs∗36) consistent with Lowe syndrome. This finding contributes to the understanding of genotype–phenotype correlations and highlights intrafamilial phenotypic variability among female carriers.

The *OCRL* gene encodes phosphatidylinositol 4,5‐bisphosphate‐5‐phosphatase, a protein localized to the trans‐Golgi network that regulates actin polymerization and plays a critical role in intracellular trafficking [27]. Although the precise mechanisms underlying Lowe syndrome and Dent Disease 2 remain incompletely defined, *OCRL* has been shown to function throughout the early endocytic pathway, including clathrin‐coated pits. Disruption at multiple sites within this pathway has been proposed as a pathogenic mechanism [28]. Erdmann et al. [29] further demonstrated that *OCRL* interacts with adaptor molecules to regulate receptor trafficking in the brain and kidneys. Moreover, studies of fibroblasts from patients with Lowe syndrome have shown impaired actin polymerization, which compromises the formation and function of tight and adherens junctions, defects that may underlie both renal pathology and CC [27]. In the present study, RNA sequencing provided additional evidence supporting the pathogenicity of the *OCRL* variant. A significant downregulation of *OCRL* expression was observed in the affected fetus, accompanied by dysregulation of genes related to actin cytoskeleton organization, actin binding, cell adhesion, endocytosis, and tight junction pathways. These findings are consistent with previous reports and support a loss‐of‐function mechanism underlying the novel *OCRL* variant.

Furthermore, our study also reveals a significant downregulation of *PITX3* gene expression in the fetus from Family 2. It is known that *PITX3* is crucial for lens formation [30]. Moreover, studies have shown that *OCRL* gene deficiency would lead to the accumulation and increased content of its substrate, phosphatidylinositol 4,5‐bisphosphate (PI(4,5)P_2_) [31]. Subsequently, PI3K may phosphorylate PI(4,5)P_2_, resulting in excessive activation of the PI3K–AKT signaling pathway, and regulate *PITX3* expression through the downstream transcription factors (such as FOXO, GSK3*β*, and mTOR). Although our RNA sequencing results indicated a significant disruption of the PI3K–AKT signaling pathway, extensive functional studies are warranted to verify this potential novel molecular mechanism.

The present study has several limitations. In Family 1, the *CRYGD* c.154 T > C (p.S52P) variant was suspected as the genetic cause of CC, but the evidence remains incomplete; additional functional studies are required to confirm its pathogenicity, such as in vivo studies using animal models. Further, genetic testing was limited to available family members, restricting the scope of segregation analysis. In Family 2, a pathogenic variant in the *OCRL* gene associated with Lowe syndrome was identified in the fetus during the most recent pregnancy. It is recommended that preimplantation genetic testing or prenatal diagnosis should be performed for this family in subsequent pregnancies. In addition, it is essential to fully inform the family that female carriers may exhibit phenotypic heterogeneity.

The present study reports, for the first time, two novel variants in the *CRYGD* and *OCRL* genes, which may be associated with CC in the Chinese families. The novel variant of c.1046dup(p.M349Ifs∗36) in *OCRL* is likely to cause Lowe syndrome through a loss‐of‐function mechanism, as supported by RNA sequencing analysis. These findings expand the mutational spectrum of CC and underscore the diagnostic utility of WES in affected individuals.

## Author Contributions

J.Z. wrote the manuscript. N.H., Y.C., and H.L. recruited participants, performed whole exome sequencing, and conducted data analysis. W.F., J.W., C.C., and J.Z. revised and edited the manuscript.

## Funding

This study was supported by the Quanzhou City Science and Technology Program of China, 2023NS068 and 2025QZC37R, and the Joint Funds for the Innovation of Science and Technology, Fujian Province, 2024Y9459 and 2023Y9255.

## Disclosure

All authors reviewed and approved the final version of the manuscript.

## Ethics Statement

Ethics Committee approval was obtained from the Institutional Ethics Committee of Quanzhou Women′s and Children′s Hospital prior to the commencement of the study (2023No.56). All patients signed written informed consent forms and agreed to the use of relevant data and information for scientific research.

## Consent

Written informed consent for publication of genetic data and relevant clinical information was obtained from all participants, including consent for publication of their children′s data.

## Conflicts of Interest

The authors declare no conflicts of interests.

## Supporting information


**Supporting Information** Additional supporting information can be found online in the Supporting Information section. As shown in File S1, RNA sequencing results elicited 2875 differential expressed genes in the fetus compared with the aged‐matched controls. Notably, a significant downregulated expression of the *OCRL* gene was observed in the fetus.

## Data Availability

The datasets generated and/or analyzed during the current study are available from the corresponding author upon reasonable request.
